# Defect Rich Hierarchical Porous Carbon for High Power Supercapacitors

**DOI:** 10.3389/fchem.2020.00043

**Published:** 2020-02-04

**Authors:** Peng Cai, Kangyu Zou, Xinglan Deng, Baowei Wang, Guoqiang Zou, Hongshuai Hou, Xiaobo Ji

**Affiliations:** ^1^College of Chemistry and Chemical Engineering, Central South University, Changsha, China; ^2^School of Materials Science and Engineering, Zhengzhou University, Zhengzhou, China

**Keywords:** high power density, hierarchical porous carbon, supercapacitors, biomass carbon

## Abstract

Tuning hierarchical pore structure of carbon materials is an effective way to achieve high energy density under high power density of carbon-based supercapacitors. However, at present, most of methods for regulating pores of carbon materials are too complicated to be achieved. In this work, a durian shell derived porous carbon (DSPC) with abundant porous is prepared through chemical activation as a defect strategy. Hierarchical porous structure can largely enhance the transfer rate of electron/ion. Furthermore, DSPC with multiple porous structure exhibits excellent properties when utilized as electrode materials for electric double layer capacitors (EDLCs), delivering a specific capacitance of 321 F g^−1^ at 0.5 A g^−1^ in aqueous electrolyte. Remarkably, a high energy density of 27.7 Wh kg^−1^ is obtained at 675 W kg^−1^ in an organic two-electrode device. And large capacity can be remained even at high charge/discharge rate. Significantly, hierarchical porous structure allows efficient ion diffusion and charge transfer, resulting in a prominent cycling stability. This work is looking forward to providing a promising strategy to prepare hierarchical porous carbon-based materials for supercapacitors with ultrafast electron/ion transport.

## Introduction

Supercapacitors (SCs) have been triggered intensive attention in energy storage due to their fast reaction dynamics, ultrahigh power density, and excellent cycling stability (Li et al., 2011; Xie et al., 2012; Hao et al., 2013; Wang and Xia, 2013; Béguin et al., 2014). Among various kinds of electrode materials for SCs, carbon materials are most promising because of their large availability, high conductivity, and cycling stability. Preparing carbon materials economically and efficiently, increasing the energy density, and enhancing the power density of carbon materials are important aspects of developing carbon materials for advanced supercapacitors (Chen X. et al., 2014; Han et al., 2014; Yang and Zhou, 2017). Since the capacity of carbon materials depends on the amounts of electrostatic charges stored at the interfaces of the electrodes and electrolytes, a lot of researches have been done to design the desired carbon materials for ions storage. Tailoring the hierarchical pore structure to match the appropriate electrolyte ions is an effective method to achieve large capacitance, efficient ion diffusion, and charge transfer (Zhu et al., 2011; Fang et al., 2012; Chen et al., 2016, 2017; Yu et al., 2016; Liu et al., 2017; Shi et al., 2017, 2019).

In recent years, some brilliant materials (Li et al., 2015, 2019a; Han et al., 2016; Cao et al., 2017; Lv et al., 2017a,b; Xu B. et al., 2017; Zhou and Hu, 2017; Zhou et al., 2018), including carbon nanotubes, grapheme, and their derivatives, have been widely explored and utilized because of their excellent thermal stability, remarkable tenacity, and high strength (Wen et al., 2016; Zhu et al., 2017; Liu et al., 2018). However, there are some obstacles in applying these materials as electrode materials for supercapacitors. The charge/discharge processes are directly affected by ionic kinetics, obviously under harsh charge/discharge conditions (current density higher than 10 A g^−1^) (An et al., 2001; Ra et al., 2009; Gao et al., 2012; Hahm et al., 2012; Sun et al., 2013; Zhu et al., 2016a). For instance, a capacitance of 362.2 F g^−1^ is obtained for the treated fullerene/graphite carbon electrodes at the low current density. However, when the current density increases to fifty times, the capacitance drops rapidly to half of the original value (Chen Z. et al., 2014; Zheng et al., 2015; Xu M. et al., 2017; Chen et al., 2018; Li et al., 2019b). It is not hard to note that increasing energy density under high power density of carbon electrodes is a great challenge. To the best of our knowledge, the poor performance in large current condition is associated with the sluggish ion migration in pores (Ye et al., 2019). It is difficult to keep good performances during charge/discharge processes without reasonable pore design. And electronic conductivity and ionic kinetics in electrodes can also be improved through regulating intrinsical structures of carbon materials (Raymundo-Piñero et al., 2011; Mun et al., 2013; Zhu et al., 2016b). In general, internal pores affect ion transport while external pores are related to ion diffusion from electrolyte. Particularly, the reduced electrode potential caused by low access to ion in pores is the main impediments under large current (Presser et al., 2011; Gao et al., 2013; Wang et al., 2015). In order to explain the importance of the pores in carbon electrode theoretically, the transmission line model is employed to imitate the charge reserve characteristic of different pore shapes, showing that porous electrodes are conducive to charge accumulation (Black and Andreas, 2010; Zhang et al., 2017). The results are proposed that electrodes with multiscale pores are beneficial to ion diffusion, which is the key to obtain large capacitance at ultra-high current density. Micropores larger than 0.7 nm are found to be used as electrolyte reservoirs to store ions (Péan et al., 2014). Under the effect of the electric field, due to the shortened length of the ion diffusion, the accumulated ions can diffuse into the nearby micropores in a shorter time than the ions in the electrolyte. And pores larger than 2 nm assist to decrease the ion confinement in the micropores, significantly enhancing the effective self-diffusion coefficient of the ions, which is advantageous for obtaining excellent rate performance (Forse et al., 2017). Therefore, a better understanding of the relationship between pore structure and capacitance performance is essential to fabricate hierarchical porous structure in carbon materials. It will accelerate ion kinetics to obtain carbon electrode materials with high ion transfer rate, which can achieve high energy storage under high current density. In order to obtain high-performance carbon electrode materials with hierarchical pore stricture, the strategy for fast ion transfer leaded by economical and efficient synthesis has been focused as main interests (Chen et al., 2013; Wang et al., 2017).

The main approaches to design hierarchical porous carbon materials are as following: (1) pores or defects generated on the surface or matrix through physical or chemical activation; (2) pores created by multidimensional carbon coupling/self-assembly; (3) adjustment of microstructures by means of templates; (4) pores congregated by the polymerization of nanoscale building blocks (Kang et al., 2015; Ni and Li, 2016; Yin et al., 2018; Kong et al., 2019). In these methods above, the size and shape of the pore array can be well-adjusted by changing the characteristics of template. However, the instability of nanoscale templates under high temperature or solvent treatment limits its extensive application (Fuertes et al., 2005; Lv et al., 2012). The process of multi-dimensional carbon coupling with the guest species and polymerization through building blocks is also tedious. Consequently, a wide range of biomass precursors combined with efficient activation methods are properly employed to prepare high-performance hierarchical porous carbon materials. Hierarchical pores in the surface would be beneficial to the transport of ions between adjacent layers, maximizing the reachable surface area. Due to the resulted multiple porous structure, the advantageous electronegativity also shows that carbon materials possess superior electrochemical performance in energy storage (Gao et al., 2019; Cai et al., 2020). Durian is widely distributed in the Southeast Asia region and around 90,000 metric tons of durian wastes were generated annually. Note that the advantages of durian shells are easy availability, cheap and high economic value. The reason of choosing durian shell as precursor to prepare carbon electrode can be attributed to main two reasons: (1) the hierarchical porous structure of the durian shell shows fast mass transfer channel, carbon material derived from durian shell precursor maintain the inherit structure features, possessing the fast ion/electron transport rate; (2) durian shell contain some heteroatoms, which can introduce defects during carbonization process, increasing the wettability of the surface of the carbon material, and promoting the contact of electrolyte area. In this work, a multi-stage porous carbon material (DSPC) is successfully obtained through the pre-carbonization and activation of the durian shell. By adjusting the activation temperature, the degree of porosity of the DSPC can be well-manipulated. The DSPC exhibits typical hierarchical porosity, reasonable aperture distribution, and high specific surface area. Surprisingly, symmetric supercapacitors based on DSPC electrodes deliver large capacity and fast ion transfer characteristics in aqueous electrolyte and organic electrolyte.

## Experimental Section

### Preparation of DSPCs

Firstly, the durian shell is cleaned several times by distilled water to remove some impurities and put into an oven under 120^o^C and for 24 h. Secondly, the dried durian shell is directly transferred to a tubular furnace in Ar atmosphere. The carbonized temperature is set to 400°C for 2 h with a heating rate of 3^o^C min^−1^. The obtained sample is marked as DSPC. Finally, the DSPC is manufactured with surface defects to obtain hierarchical channels. DSPC is mixed and grinded with KOH of 4:1 mass ratio. In order to explore the most appropriate the ratio of KOH to C, the corresponding electrochemical results are obtained in Figure S1. They are divided into five parts for subsequent activation and immediately transferred to a tubular furnace for generating surface defects. The temperature is 600, 700, 800, 900, and 1,000°C for 1 h. All the samples after being calcined are added 6 M HCl until pH = 7. All samples are then washed to neutral and dried in an oven. The carbon materials activated under different temperature are denoted as DSPC-600, DSPC-700, DSPC-800, DSPC-900, and DSPC-1000, respectively.

### Material Characterization

The structure of DSPCs are explored by X-ray diffraction (XRD) with a scan rate of 10°C min^−1^ by using Kα radiation of Cu (λ = 0.1542 nm). Degree of graphitization is measured through Raman spectroscopy (DXR, Thermo-Fisher Scientific). The morphology of all the samples are investigated by field-emission scanning electron microscopy (FESEM, JSM-7600F, JEOL) and transmission electron microscopy (JEM-2010F at 200 kV, JEOL). The specific surface area is acquired from the N_2_ adsorption-desorption isotherms and is obtained by the Brunauere Emmette Teller (BET) equation. The chemical bond of DSPCs are collected from X-ray photoelectron spectrometer (XPS) instrument.

### Electrochemical Characterization

All the samples are measured on a three-electrode system using aqueous electrolyte and on two-electrode system using aqueous electrolyte and organic electrolyte for determining their capacitive performance.

For three-electrode aqueous electrolyte system, every carbon samples are grinded with carbon black and polytetrafluoroethylene (PTFE) with a weight ratio of 8:1:1 loaded on nickel foam. The electrode plates are then dried in a vacuum at 80°C for 12 h. Pieces are pressed under 10 MPa for 1 min. The mass load of each electrode plate is 2.2 mg/cm^2^. Platinum electrode is selected as the counter electrode, and a saturated calomel electrode is selected as the reference electrode. The electrolyte is 6 M KOH.

For two-electrode aqueous electrolyte system, every carbon samples are grinded with carbon black and polytetrafluoroethylene (PTFE) with a weight ratio of 8:1:1 loaded on nickel foam. The electrode plates are then dried in a vacuum at 80°C for 12 h. Pieces are pressed under 10 MPa for 1 min. The electrolyte is 6 M KOH. CR2016-type coin cells are assembled accordingly. Electrochemical test begins after several hours.

For two-electrode organic electrolyte system, each carbon samples are grinded with carbon black and polyvinylidene fluoride (PVDF) with a weight ratio of 8:1:1 loaded on Al foils. The electrode plates are then dried in a vacuum at 120°C for 12 h. CR2016-type coin cells are assembled inside the Braunglovebox with argon atmosphere (<0.1 ppm of both O_2_ and H_2_O). Galvanostatic charge/discharge (GCD) tests are carried out between 0 and 2.7 V by an Arbin BT2000 instrument. The cyclic voltammetry (CV) tests are carried out at different scan rates on a Solartron 1470 Multistat system. The electrochemical impedance spectroscopy (EIS) measurements are performed using an electrochemical workstation.

The gravimetric capacitance is calculated from the CV curves and discharge curves, according to the formula:

$$\begin{array}{l} Cs=\frac{I\Delta t}{m\Delta V} \end{array}$$

where *Cs* is the specific capacitance and Δ*V* is the voltage drop. Δ*t* is the discharging time. *I/m* is referred to the current density.

The power density and energy density of the devices are obtained thorough using the following equations:

$$\begin{array}{l} P=\frac{△V\times I}{2\times m}\\ E=\frac{P*t}{3600} \end{array}$$

Here, Δ*V* is referred to the discharge voltage, *I* is referred to the discharge current, *t* is referred to the discharge time and m is referred to the total mass of active materials, respectively.

## Results and Discussion

**Figure 1:**
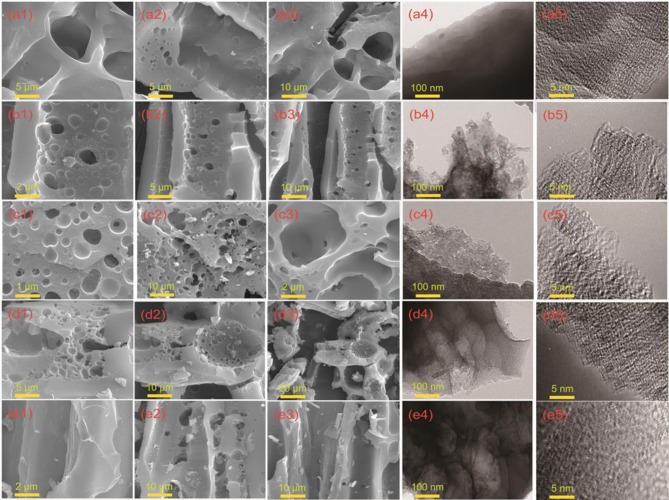
Morphology and structure characterization of DSPCs. **(a1-a3)** SEM image of DSPC-600, **(a4,a5)** TEM and HRTEM image of DSPC-600. **(b1-b3)** SEM image of DSPC-700, **(b4,b5)** TEM and HRTEM image of DSPC-700. **(c1-c3)** SEM image of DSPC-800, **(c4,c5)** TEM and HRTEM image of DSPC-800. **(d1-d3)** SEM image of DSPC-900, **(d4,d5)** TEM and HRTEM image of DSPC-900. **(e1-e3)** SEM image of DSPC-1000, **(e4,e5)** TEM and HRTEM image of DSPC-1000.

SEM and TEM images of hierarchical porous carbon materials prepared from the durian shell are shown in Figure 1 and Figure S2. It is obvious that the pores are hardly manufactured at 600°C. When the temperature is increased to 700°C, the pore structure in the carbon material is gradually generated, and an uneven structure is produced on the surface. As the temperature is further risen up to 800°C, the pore structure formed in the carbon material are widely ranged and the structure is uniform. When the temperature is raised to 900°C, the defect of the surface is degenerated and destroyed, and the pore structure of the material is broken. As the temperature is kept at 1,000°C, the structure of hierarchical pores is collapsed more severely, and the surface defect structure is difficult to be observed. According to TEM images, an amorphous dominated porous structure and defect architecture are further revealed.

**Figure 2:**
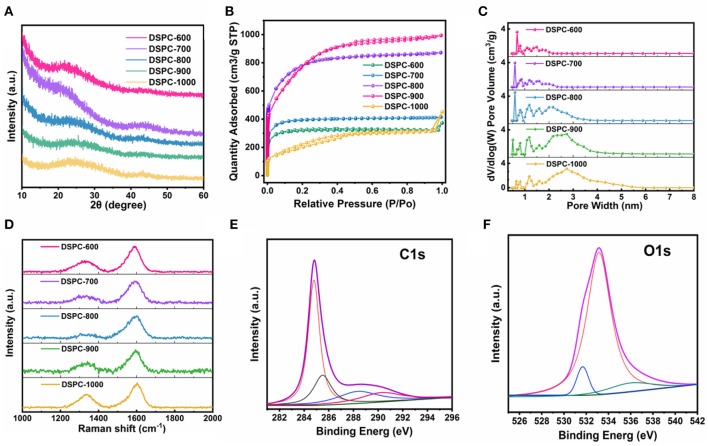
**(A)** XRD patterns, **(B)** N_2_ adsorption-desorption isotherms, **(C)** Pore size distribution, and **(D)** Raman spectrum of DSPCs. **(E,F)** high-resolution XPS C1s and O1s spectra for DSPC-800.

The XRD measurement of all samples are exhibited in Figure 2A. The broad peaks located at 23° and inconspicuous peaks centered at 43° are perceived, indicating that all the samples are in amorphous constructions maintained few regions of crystallinity (Hou et al., 2015, 2017; Ge et al., 2018; Zou et al., 2018; Wu et al., 2019). More detailer pore structures of the samples are collected by the nitrogen adsorption/desorption measurements in Figures 2B,C. The isotherms of all DSPCs exhibit a combination of I-type and IV-type isotherm characteristics at a relative pressure >0.4, suggesting that both micropores and mesopores exist. Notably, the specific surface area of DSPC-600, DSPC-700, DSPC-800, DSPC-900, and DSPC-1000 are 1006, 1217, 2535, 2602, and 899 m^2^ g^−1^, respectively. When the temperature is increased slightly, the surface area of micropores and mesoporous increase. As the increase of temperature, the proportion of micropores decreases while the proportion of mesopores increases according to the higher platform and lower starting angles of the low-pressure areas of DSPC-900 in Figure 2B. The plots of pore size distributions are revealed the porous structure for all the samples. Furthermore, wide existence of nanopores in plots of pore size distributions illustrates hierarchical porous structure of DSPC-800. Risen from 800°C to 1,000°C, the distribution of micropores gradually decreases and the distribution of mesopores becomes larger. In general, as depicted in Figure 2C, the DSPC-800 exhibits a hierarchically aperture and porosity structure. The Raman spectra of the acquired samples are deconvoluted into two peaks including G peak and D peak, and the intensity ratio of G band vs. D band (IG/ID) can be used to describe the degree of graphitization. The G peak is induced by Sp2-hybrid carbon and D peak is related to the disordered graphite structure. As shown in Figure 2D, the values of IG/ID of all samples are 1.65, 2.52, 6.6, 2.06, and 1.59, respectively, demonstrating the high graphitization degree of DSPC-800. Note that Sp2 hybridization-dominant carbon is widely employed to improve ion transfer rate and efficient energy storage because of large graphitization, high electrical conductivity, and thermal stability. Benefiting from high value of IG/ID in DSPC-800 superior kinetics performance during chare/discharge process could be delivered. Furthermore, the surface chemical environment of DSPC-800 is determined in details by XPS analysis. Carbon content in the DSPC-800 is 91.02 at%, and the oxygen content of the DSPC-800 is 8.98 at %. As shown in Figure 2E, the C1s profiles is divided into four peaks of COOH (290 eV), C = O (286.7 eV), C-O (285–286.eV) and C = C/C-C (284.6 eV). The high-resolution O1s spectra can be deconvoluted into three peaks located at 531.0, 533, and 536 eV, which is corresponding to the C=O, C-O, and carbonyl (Figure 2F), respectively (Yang et al., 2019; Zhu et al., 2020).

**Figure 3:**
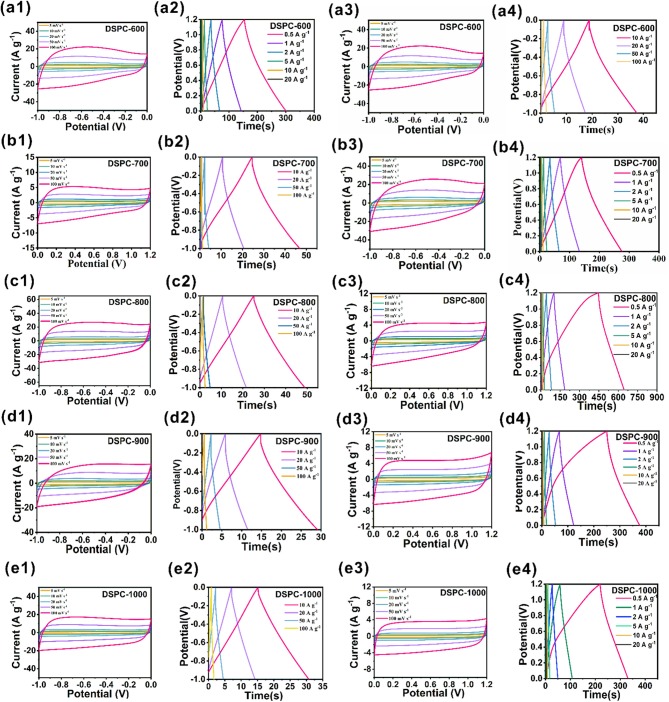
Electrochemical performances of DSPCs. **(a1–e1)** CV curves at three electrode system in 6 M KOH, **(a2–e2)** charge/discharge curves at three electrode system in 6 M KOH, **(a3–e3)** CV curves at two electrode system in 6 M KOH, **(a4–e4)** charge/discharge curves at two electrode system in 6 M KOH.

The capacitive performance of DSPCs are firstly measured in 6.0 M KOH/H_2_O electrolyte by using a three-electrode system. The CV curves of all the samples exhibit quasi-rectangular appearance at the scan rate of 5–100 mV s^−1^, implying an ideal capacitive characteristic in Figures 3a1–e1. The DSPC-800 shows the largest area of CV curves among all the samples, suggesting outstanding charge storage feature. In addition, according to the results in Figure S1, the ratio of KOH to C should be selected as 4: 1 rather than 3:1 or 5:1. As displayed in Figure 3c2, the GCD curve of DSPC-800 presents the longest discharge time among the five samples at the current density of 10 A g^−1^, suggesting the largest capacitance among all the samples. Based on the results of GCD curves, the specific capacitances of DSPC-600, DSPC-700, DSPC-800, DSPC-900, and DSPC-1000 are 239.5, 336.6, 320.5, 209.6, and 186 F g^−1^ at a current density of 0.5 A g^−1^ (Figures 3a2,b2,d2,e2), exhibiting that the specific capacitances of DSPCs are 194.1, 249.1, 253.1, 158.9, and 159 at 5 A g^−1^, respectively. Impressively, the specific capacitances of DSPCs are 130.4, 93.7, 154.75, 50, and 60 F g^−1^ at a current density of 50 A g^−1^, separately. The capacitive performances of DSPCs are subsequently evaluated in 6.0 M KOH/H_2_O electrolyte using a two-electrode system. The CV curves of the device are listed in Figure 3c3, in which the quasi-rectangular appearance of DSPC-800 is kept even at the scan rate of 100 mV s^−1^, demonstrating an excellent rate performance. This result is further illustrated by the GCD curves at different current densities in Figures 3a3–e3. When the current density is 0.5 A g^−1^, the specific capacitances of DSPCs devices are determined as 56.1, 60.9, 82, 52, and 46.3 F g^−1^, respectively, corresponding to the specific capacitances of 45.1, 42.7, 63.3, 33, and 32 F g^−1^ at 5 A g^−1^, respectively. As the current density increases to 20 A g^−1^, specific capacitances of 30.2, 25.1, 32.1, 9.3, and 12 F g^−1^ are achieved for DSPCs samples. The highest specific capacitance of DSPC-800 is attributed to hierarchical porous structure, which can bring numerous active sites for the charge storage in Figures 3a4–e4.

**Figure 4:**
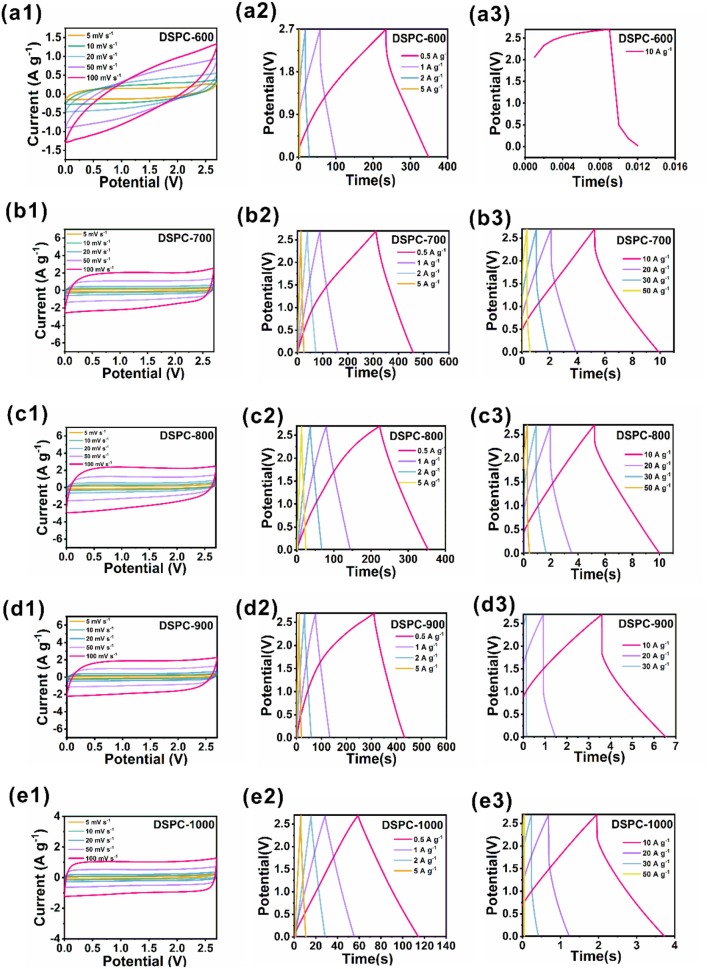
Electrochemical performances of DSPCs. **(a1–e1)** CV curves at two electrode system in organic electrolyte, **(a2–e2)** charge/discharge curves at two electrode system in organic electrolyte, **(a3–e3)** charge/discharge curves at two electrode system tested in large current in organic electrolyte.

All the samples are further tested in organic electrolyte in two-electrode devices based on DSPCs. The device of DSPC-600 measured in organic electrolyte is shown an obvious polarization, which is attributed to sluggish ion kinetics behavior of the electrode (Figure 4a1). The low energy density of the device of DSPC-600 is 0.01 Wh kg^−1^, which is suggested andante charge transport and ion diffusion in large current density shown in Figure 4a3. The low capacity can be attributed to the less surface defects and low conductivity due to the low activation temperature. The other four devices are shown with a quasi-rectangular shape CV curve that can be interpreted as the presence of higher surface defects. The DSPC-800 device shows a highly symmetric shape, suggesting great electrochemical reversibility (Figures 4b1–e1). It is important to noted that the device of DSPC-800 exhibits smallest IR drop at 10 A g^−1^, implying that surface defect structure with relatively high hierarchical aperture and porosity can facilitate transfer rate of the electrolyte ions and decrease the ionic diffusivity resistance. This result is further illustrated by the GCD curves at different current densities (Figures 4a3–e3). The energy density of all the devices are 21.5, 24.3, 27.7, 22, and 10.4 Wh kg^−1^ at a power density of 675 W kg^−1^, respectively. Furthermore, energy densities of 0.6, 20.7, 21.5, 16.4, and 8.4 Wh kg^−1^ can be obtained for related samples at a power density of 6,750 W kg^−1^. Surprisingly, even at 67,500 W kg^−1^, the device of DSPC-800 can also deliver an energy density of 4.2 Wh kg^−1^. The highest energy density of the devices with DSPC-800 as electrode are attributed to multiple pores that can supply numerous active sites for the charge storage in Figure 4. It is clear that hierarchical porous structure can effectively improve the electrochemical properties of materials in both aqueous electrolyte and organic electrolyte system, suggesting that the introduction of hierarchical porous structure is significant.

**Figure 5:**
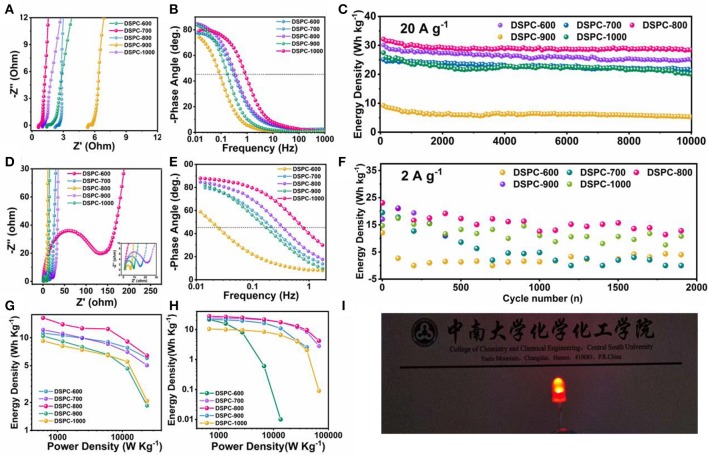
**(A)** EIS analysis in 6 M KOH, **(B)** Bold plot in 6 M KOH, **(C)** cycle performance in 6 M KOH, **(D)** EIS analysis in organic electrolyte, **(E)** Bold plot in organic electrolyte, **(F)** cycle performance in organic electrolyte, **(G,H)** Ragon plot in 6 M KOH and organic electrolyte, **(I)** optical picture of device of DSPC-800 in organic electrolyte.

A number of electrochemical measurements including cycling stability and electrochemical impedance spectroscopy (EIS) are carried out to investigate the electrochemical performance of DSPCs. Low resistance and rate capability are determined by the efficiency of charge transport and ion diffusion in aqueous electrolyte system (Xu et al., 2014). The contact resistance between the electrode material and the electrolyte also affects resistance (Rs). Because the ion transfer characteristics of these synthesized materials are different, Rs is generally different (Jin et al., 2018a,b; Nawwar et al., 2019; Wang and Cui, 2019; Yuan and Lu, 2019). The surface defect of DSPCs is critical for achieving such high specific capacitance. EIS analysis is revealed that DSPC-800 exhibits an ignorable combined series resistance, which is greater than other samples. The steep slope of the Nyquist plot in the low frequency domain indicates fast ion transport in the surface defect electrode. The lack of semicircle of DSPC-800 in the middle frequency domain further suggests that the charge transfer resistance (Rct) at the electrode/electrolyte interface is negligible (Figure 5A). In organic electrolyte system, DSPC-800 also exhibits a small resistance, which is similar to the results in aqueous electrolyte system in Figure 5A. The large resistance of DSPC-600 is correlated with previous results. Bode phase diagram also discloses some important information in Figures 5B,E. Firstly, the 0.1 Hz phase angle is approximately the ideal phase angle, that is −90°, and the ideal phase angle is closed to the ideal capacitive behavior. Secondly, the characteristic frequency (f_0_) is corresponded to a −45° phase angle. The characteristic time constant can be expressed as: τ_0_ = 1/f_0_. The smaller τ_0_ represents the faster frequency response and τ_0_ is related to the rate characteristics. Bode phase diagram is revealed that surface defect is contributed to ion diffusion (Figures 5B,E). A high capacity residue of 88.71% can be acquired for DSPC-800 in aqueous electrolyte system at 20 A g^−1^ after 10,000 cycles. The capacity retentions of other devices are 82.9, 84.75, 57.64, and 72.98% in aqueous electrolyte system at 20 A g^−1^ (Figure 5C), respectively. DSPC-800 is also preserved good cycle performance in organic electrolyte system after several thousand times (Figure 5). Note that carbon materials with hierarchical porous structure assembled devices can deliver remarkable performance and show practical applications (Figures 5G–I).

## Conclusion

Here, defect strategy is demonstrated as an effective method to construct hierarchical porous structure with fast ion transport property. The hierarchical porous structure provides larger surface area for the infiltration of electrolyte for ions transport into interior multiple pores. Consequently, hierarchical porous structure can optimize ion kinetics by affecting ion transport of internal pores and ion diffusion of external pores from electrolyte, especially at large current density. Due to the hierarchical porous structure tuned high ionic conductivity and optimized ionic kinetics of electrodes, the power performance is impressive, delivering excellent gravimetric capacitance both in aqueous and organic electrolyte. This work provides a bright and effective approach to fabricate the carbon materials with hierarchical porous structure. Surprisingly, it has been successfully applied in energy storage under large current density. Considering the advantages of low cost and abundance, hierarchical porous carbon is extremely promising for the development of advanced electrode materials in super capacitors.

## Data Availability Statement

The raw data supporting the conclusions of this article will be made available by the authors, without undue reservation, to any qualified researcher.

## Author Contributions

PC and GZ designed the research, performed the experiments, and made contributions to the acquisition, analysis, and interpretation of data for the work. KZ, XD, and BW carried out the partial experimental characterization. HH and XJ revised this article and approved of the version to be published. All authors incorporated in the discussion of experimental results.

### Conflict of Interest

The authors declare that the research was conducted in the absence of any commercial or financial relationships that could be construed as a potential conflict of interest.
