# Supplementary material for: Defect Rich Hierarchical Porous Carbon for High Power Supercapacitors
Source: Front Chem. 2020 Feb 4;8:43. doi: 10.3389/fchem.2020.00043 (PMC7011847; doi:10.3389/fchem.2020.00043)
Supplement: Supplementary file 1 [file Data_Sheet_1.pdf]

Supporting Information

Defect Strategy Generated Hierarchical Porous Carbon for High Power Supercapacitors

Peng Cai, Kangyu Zou, Xinglan Deng, Baowei Wang, Guoqiang Zou*, Hongshuai Hou, and Xiaobo Ji

State Key Laboratory of Powder Metallurgy, College of Chemistry and Chemical Engineering, Central South University, Changsha, China410083,

*Correspondence:

gq-zou@csu.edu.cn

Part I: Supplementary Figures and tables

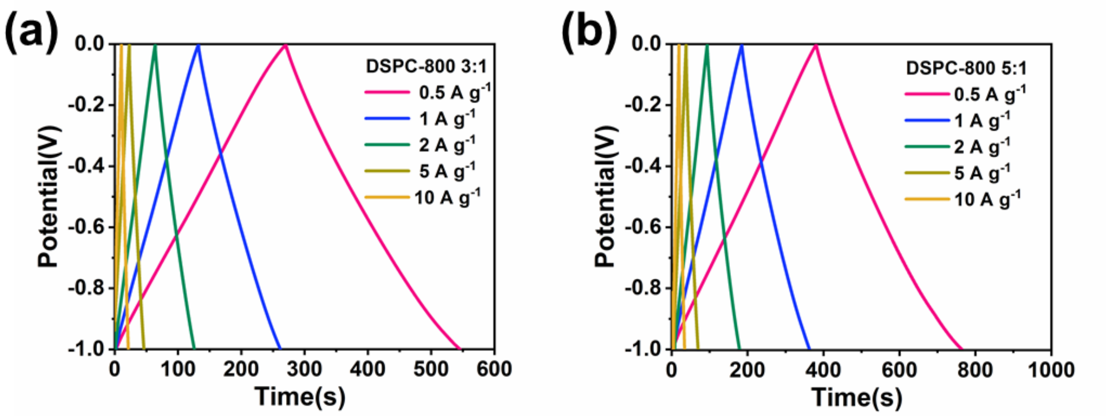

Figure S1. GCD curves of DSPC activated in 800 ^{o}C with a different weight ratio of KOH to C in 6.0 M KOH electrolyte. (a) weight ratio of 3 : 1; (b) weight ratio of 5 : 1.

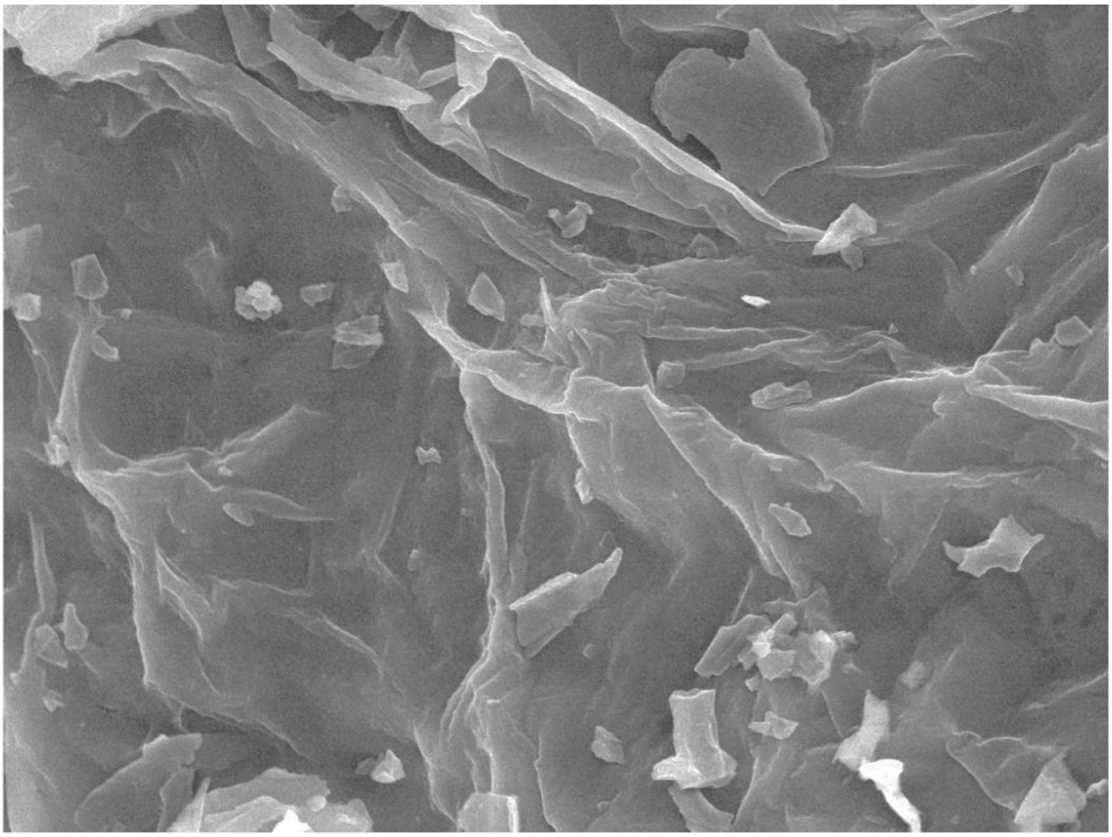

Figure S2. SEM image of DSPC
